# The Prevalence of Psychiatric Disorders Among Elective Plastic Surgery Patients

**Published:** 2019-03-18

**Authors:** Benjamin Jang, Dhaval R. Bhavsar

**Affiliations:** ^a^University of Kansas School of Medicine, Kansas City, KS; ^b^MacNeal Hospital, Berwyn, IL

**Keywords:** psychiatric, cosmetic surgery, plastic surgery, anxiety, depression

## Abstract

**Purpose:** Psychiatric disorder is one of the predictors of poor outcome in cosmetic plastic surgery patients. A US study in 1960 showed that 72.4% of 98 cosmetic plastic surgery patients had a psychiatric disorder. In our study, we predict that the prevalence of psychiatric disorders will be statistically significant among patients seeking elective plastic surgery in comparison with the general US population. **Methods:** We conducted a retrospective review study of 1000 adult patients seeking elective plastic surgery at The University of Kansas Medical Center Plastic Surgery Department from 2011 to 2016. **Results:** From 1000 patients seeking elective plastic surgery procedure, 441 (44.1%) patients have or had a history of psychiatric disorder. Most common psychiatric disorders were major depressive disorder (*n* = 223; 50.6%) and generalized anxiety disorder (*n* = 145; 32.9%). **Conclusion:** Our study indicates that psychiatric disorders are prevalent in patients seeking elective plastic surgery at our institution. Anxiety and depression were the most common diagnoses, and this is possibly due to these being the most common psychiatric disorders in the US population. Results highlight the importance of provider vigilance for psychiatric patients seeking elective plastic surgery.

To have successful cosmetic plastic surgery result, it is imperative to assess candidates for predictors of poor outcomes. These include the following: psychiatric disorder, demographic factors (male and younger age), relationship issues, unrealistic expectations, previous dissatisfied surgery, and minimal deformity.^1^ For psychiatric patients, despite having technically satisfactory cosmetic surgery, poor emotional adjustment and social functioning were seen post procedure. These symptoms included poor self-image, relationships, and general quality of life.^1^ There are a few studies that looked at the relationship between psychiatric disorders and cosmetic plastic surgery patients. The most recent 1960 US study demonstrated that 72.4% of 98 cosmetic plastic surgery patients had an underlying psychiatric disorder.^2^ However, Hasan^3^ in 2000 commented that the correlation between the two is not as well established.

The aim of this study was to assess the current prevalence of psychiatric disorders among elective plastic surgery patients, which includes cosmetic and noncosmetic plastic surgery. We predict that the prevalence of psychiatric disorders will be significantly greater among patients seeking elective plastic surgery than in the general US population. This study gives an updated view of the 1960 literature in the United States by having a larger sample size, inclusion of noncosmetic plastic surgery patients, and comparison of results with national data. We also compare the characteristics of patients with and without psychiatric disorder seeking elective plastic surgery.

## METHODS

We conducted an institutional review board–approved retrospective review of 1000 adult patients (female: *n* = 859; male: *n* = 141) seeking elective plastic surgery at The University of Kansas Medical Center Plastic Surgery Department from 2011 to 2016. Psychiatric disorders were determined by examining patient charts for their history of diagnoses and psychiatric medications. Patients at least 18 years of age coming in for elective plastic surgery consult were included in this study. Notable exceptions to elective plastic surgery included cancer or suspicious for cancer excision, hidradenitis suppurativa excision, scar revision, keloid removal, breast reconstruction immediately after breast cancer surgery, trauma, and hand pathologies. These elective cases included 803 cosmetic plastic surgery patients, with cosmetic plastic surgery categorized by 7 areas of interventions. First included breast: breast augmentation, breast lift, breast reduction, and fat transfer breast augmentation. Second included fat reduction: liposuction and nonsurgical fat reduction. Third included body lifts: arm lift, body contouring, body lift, buttock augmentation, mommy makeover, thigh lift, and tummy tuck. Fourth included face and neck: brow lift, chin surgery, ear surgery, eyelid surgery, facelift surgery, facial implants, neck lift, and rhinoplasty. Fifth included minimally invasive procedures: Botox, chemical peel, dermabrasion, dermal fillers, laser hair removal, laser skin resurfacing, microdermabrasion, skin rejuvenation and resurfacing, and spider vein treatment. Sixth included male-specific plastic surgery: gynecomastia surgery, hair transplant, and other male plastic surgeries. Seventh included vaginal rejuvenation: nonsurgical and surgical vaginal rejuvenation.

We performed χ^2^ test to determine the significance of psychiatric disease prevalence, types of plastic surgery performed, gender distribution, substance use, and body mass index (BMI).

## RESULTS SUMMARY

### Prevalence of psychiatric disorder

From 1000 elective patients, 441 (44.1%) patients had or have a history of psychiatric disorder. Some of these diagnoses were comorbid, as a patient could have had or has 1 or more psychiatric disorders. When compared with the general US population (26.2% of 9282 individuals per Kessler et al^4^), elective and its subgroups noncosmetic and cosmetic patients were all significantly higher in prevalence as shown in Table 1.

In Table 2, psychiatric diagnoses were determined by documented diagnosis with corresponding psychiatric medication, documented diagnosis, or psychiatric medication with a high suspicion for the diagnosis. Some of the patients with medications such as selective serotonin reuptake inhibitor (SSRI) without a documented diagnosis could have used the medication for depression or anxiety, so they were not given a specific diagnosis but instead marked for having a psychiatric disorder. Some patients taking antipsychotic medications without documented diagnosis were not given specific diagnosis either because of its many uses. In addition, someone with a documented bipolar diagnosis but taking SSRI was categorized having as psychiatric disorder without specific bipolar diagnosis for a conflicting reason. The most commonly documented psychiatric diagnoses are noted in Table 2.

### Cosmetic procedures

**Figure 1:**
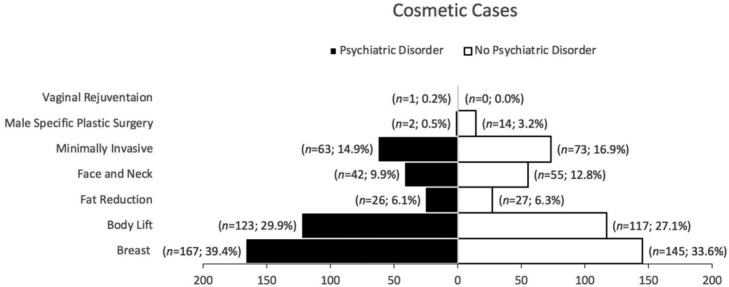
Distribution of cosmetic cases.

The distribution of cosmetic cases (*n* = 803) is shown in Figure 1. There were a total of 424 procedures in patients with psychiatric disorder and 431 procedures in patients without psychiatric disorder because a patient could receive multiple different procedures in a single encounter. Breast and body lift procedures were most common between the 2 patient populations and vaginal rejuvenation being the least common. No significance was found between the 2 patient populations except for male-specific plastic surgery (all gynecomastia), which was significantly higher in patients without psychiatric disorder (*P* = .0035).

### Gender

Regardless of the psychiatric disorder presence, the study sample consisted of a significant (*P* < .0001) amount of more female patients (*n* = 859; 85.9%) than male patients (*n* = 141; 14.1%). Women (*n* = 390; 88.4%) were also found to have a significant (*P* = .0415) higher level of psychiatric disorder than men (*n* = 51; 11.6%).

### Substance use

**Figure 2:**
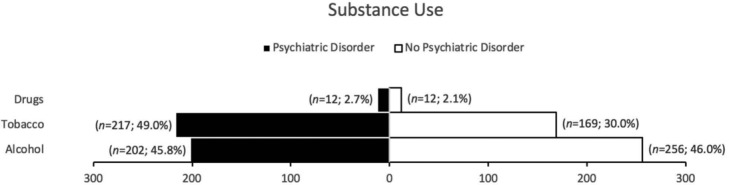
Substances usage among patients seeking elective plastic surgery procedure.

Figure 2 shows the substance usage among 559 patients without psychiatric disorder and 441 patients with psychiatric disorder. Only tobacco usage was demonstrated to be significant (*P* < .0001) in patients with psychiatric disorder than those without.

### Body mass index

**Figure 3:**
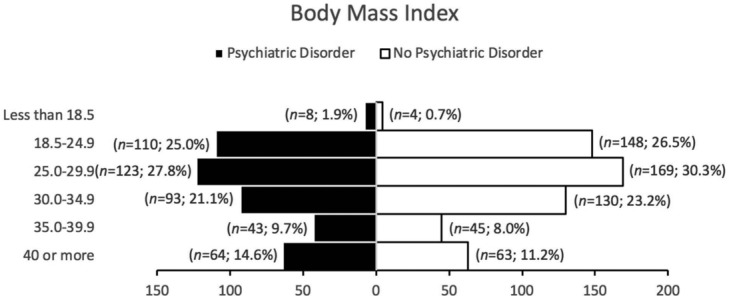
Body mass index distribution.

The average BMI among patients with and without psychiatric disorders was 31.1 and 30.3, respectively. There was no significant difference between the 2 groups. In Figure 3, the most common group includes overweight individuals with BMI between 25.0 and 29.9 and the group with least common BMI includes underweight individuals with BMI less than 18.5.

## DETAILED RESULTS AND DISCUSSION

In our institution, psychiatric disorders are prevalent in cosmetic plastic surgery patients. Like the general US population, depression and anxiety were found to be the most common diagnoses.^4^ It appears that psychiatric disorders are less widespread among US cosmetic plastic surgery patients than in those in 1960.^2^ However, this could be attributed to patients not having a prior psychiatrist evaluation as demonstrated by Edgerton et al.^2^ This could have led to an underestimation of prevalence and psychiatric diagnoses such as body dysmorphic disorder being missed and undocumented. Conversely, prevalence could be falsely elevated because of patients taking psychoactive medications such as SSRI for nonpsychiatric disorder such as menopause. Other limitations include how Kessler et al^4^ surveyed the general US population and obtained their psychiatric history differently from that in our study. It should also be noted that psychiatric diagnoses in our study were made by different individuals, specialties, and level of providers. Nonetheless, the results highlight the prevalence of psychiatric disorders among cosmetic plastic surgery patients and the need for mental illness vigilance by plastic surgeons. In addition, although Edgerton et al^2^ only looked at cosmetic plastic surgical procedures, our study suggests that psychiatric evaluation may be helpful to broader area of patients. We found psychiatric disorders to be pervasive among noncosmetic plastic surgery patients. Ultimately, it is the provider's clinical judgment to determine whether a psychiatric patient will benefit from plastic surgery. Some patients could have reversible psychiatric symptoms secondary to physical deformity.^2^ Finally, since our study involved a single academic medical center that primarily serves the Kansas City metropolitan area, there is a question of external validity. These results should be interpreted with caution to avoid unintentional bias against patients with a psychiatric history.

We also examined the characteristics of patients with and without psychiatric disorder. Among the cosmetic cases, we were not able to differentiate the psychiatric disorder prevalence by specific procedures but rather areas of concern due to lack of documentation. Our results showed that both patient groups had similar distributions of cosmetic procedures. This similarity in areas of concern and degree of intervention invasiveness suggest the lack of difference in their body image perception. However, men without psychiatric disorder had higher cases of male specific plastic surgery. Although low sample size, this could be related to psychiatric disorders being less prominent in men than women in our study; similarly, this type of prevalence pattern can be seen in general population.^5^ As for substance use, psychiatric patients were noted to have higher consumption of tobacco products than non-psychiatric patients. This usage disparity has also been noted in general population and is likely associated with patients' efforts to regulate their psychiatric symptoms.^6^ It is important for providers to be aware of this heightened use as smoking is associated with poor outcome for cosmetic surgery and could be underreported by patients.^7^^,^^8^ Finally, the average BMIs for both groups were considered obese as they were 30 or greater. Although no significant distinction can be made between the 2 groups, obese BMI is a risk factor of morbidity and mortality for cosmetic surgery patients. Providers should be aware of patients’ body habitus regardless of their psychiatric status.^9^^,^^10^

## CONCLUSION

Psychiatric disorders are prevalent in elective plastic surgery patients, and providers should be vigilant. It is up to the clinician to determine whether the patient will benefit from plastic surgery.

## Figures and Tables

**Table 1:** Psychiatric disorder prevalence; some of these diagnoses were comorbid

	Elective (*n* = 1000), *n* (%)	Noncosmetic elective (*n* = 197), *n* (%)	Cosmetic (*n* = 803), *n* (%)
No psychiatric disorder	559 (55.9)	131 (66.5)	428 (53.5)
Psychiatric disorder	441 (44.1)	66 (33.5)	375 (46.7)
Chi-square test in comparison with national data	*P* < .0001	*P* = .02	*P* < .0001

**Table 2:** Most notable psychiatric disorder distribution*

	Elective (*n* = 441), *n* (%)	Noncosmetic elective (*n* = 66), *n* (%)	Cosmetic (*n* = 375), *n* (%)	% in total subjects (*n* = 1000)
Major depressive disorder	223 (50.6)	36 (54.5)	187 (50.0)	22.3
Generalized anxiety disorder	145 (32.9)	20 (30.3)	125 (33.3)	32.9
ADD/ADHD	31 (7.0)	8 (12.1)	23 (6.1)	3.1
Panic disorder	11 (2.5)	1 (1.5)	10 (2.7)	1.1
PTSD	11 (2.5)	1 (1.5)	10 (2.7)	1.1
Bipolar	10 (2.3)	1 (1.5)	9 (2.4)	1.0

*ADD indicates attention-deficit disorder; ADHD, attention-deficit/hyperactivity disorder; and PTSD, posttraumatic stress disorder.
